# Speech‐based digital cognitive assessment for clinical trials: Detecting cognitive impairment stages and AD biomarker relations across European cohorts

**DOI:** 10.1002/alz.71462

**Published:** 2026-05-18

**Authors:** Alexandra König, Johannes Tröger, Elisa Mallick, Nicklas Linz, Craig Ritchie, Sarah Gregory, Matthew Hunter, Kay Johnson, Gonzalo Sánchez Benavides, Oriol Grau‐Rivera, Andreea Radoi, Claudia Porta‐Mas, Stefanie Köhler, Stefan Teipel, Oskar Hansson, Pontus Tideman, Anika Wuestefeld, Sebastian Palmqvist

**Affiliations:** ^1^ ki:elements GmbH Saarbrücken Germany; ^2^ Cobtek (Cognition‐Behaviour‐Technology) Lab FRIS‐University Côte d'azur Nice France; ^3^ Centre Hospitalier et Universitaire, Clinique Gériatrique du Cerveau et du Mouvement, Centre Mémoire de Ressources et de Recherche Université Côte d'Azur Nice France; ^4^ Scottish Brain Science Edinburgh UK; ^5^ Brain Health and Neurodegenerative Medicine University of St. Andrews St. Andrews UK; ^6^ Edinburgh Dementia Prevention University of Edinburgh Edinburgh UK; ^7^ Barcelonaβeta Brain Research Center (BBRC) Pasqual Maragall Foundation Barcelona Spain; ^8^ IMIM (Hospital del Mar Medical Research Institute) Barcelona Spain; ^9^ Centro de Investigación Biomédica en Red de Fragilidad y Envejecimiento Saludable Instituto de Salud Carlos III Madrid Spain; ^10^ Servei de Neurologia Hospital del Mar Barcelona Spain; ^11^ Deutsches Zentrum für Neurodegenerative Erkrankungen (DZNE) Rostock Germany; ^12^ Department of Psychosomatic and Psychotherapeutic Medicine University of Rostock Rostock Germany; ^13^ Clinical Memory Research Unit Department of Clinical Sciences Lund University, Malmö Lund Sweden; ^14^ Memory Clinic Skåne University Hospital Malmö Sweden

**Keywords:** Alzheimer's disease, cerebrospinal fluid biomarkers, mild cognitive impairment, remote cognitive assessment, speech‐based digital biomarkers, subjective cognitive decline

## Abstract

**INTRODUCTION:**

Early detection of Alzheimer's disease (AD) is critical for timely intervention as disease‐modifying treatments emerge. Speech‐based digital biomarkers offer scalable options for remotely capturing speech‐derived functional changes associated with early cognitive decline, but validation across real‐world populations remains limited.

**METHODS:**

We evaluated the speech biomarker for cognition (SB‐C), an automated speech‐derived measure associated with cognitive status, in 736 participants across five European cohorts (Barcelonaβeta Brain Research Center's Alzheimer's at‐risk cohort, European Prevention of Alzheimer's Dementia Scotland, Dementia Study of Cognitive and Biomarker Dynamics, Longitudinal Cognitive Impairment and Dementia Study, and Biomarkers for Identifying Neurodegenerative Disorders Early and Reliably [BioFINDER‐Primary Care]). Participants completed verbal learning and semantic fluency tasks via automated phone or app‐based platforms. SB‐C performance was compared to Mini‐Mental State Examination, Clinical Dementia Rating, Preclinical Alzheimer Cognitive Composite 5, and cerebrospinal fluid amyloid beta and phosphorylated tau181 biomarker status.

**RESULTS:**

SB‐C significantly differentiated cognitively unimpaired and impaired groups (*P* < 0.001), correlated with standard cognitive measures, and showed moderate‐to‐high area under the curve (0.56–0.82) for classifying biomarker positivity, with strongest results in BioFINDER‐Primary Care.

**DISCUSSION:**

SB‐C is a scalable, remote speech‐derived marker associated with cognitive status and AD biomarker group differences.

## Highlights

- The speech biomarker for cognition (SB‐C) detects early Alzheimer's disease (AD)‐related cognitive impairment remotely.
- The SB‐C was validated across five European cohorts with diverse cognitive and biomarker profiles.
- SB‐C scores associated with cerebrospinal fluid amyloid beta and phosphorylated tau181 biomarker positivity.
- Significant differences were observed in cognitively unimpaired individuals with subjective cognitive decline by amyloid/tau status.
- SB‐C supports scalable screening for AD in decentralized clinical trial settings.

## 1 BACKGROUND

The first amyloid‐targeting antibody treatments for Alzheimer's disease (AD) have been approved in the United States, European Union, Japan, China, and elsewhere. These treatments are most effective when administered in the earliest symptomatic stages of the disease, such as mild cognitive impairment (MCI) or mild dementia, which makes early detection of cognitive impairment important.^1^

Dementia affects > 55 million people worldwide and prevalence is rising, with MCI representing a key prodromal stage.^2^, ^3^, ^4^ Many individuals with early cognitive impairment remain undiagnosed,^5^ limiting access to disease‐modifying treatments (DMTs).^6^

Traditional methods for diagnosing MCI, such as brief cognitive tests administered in primary care settings, are not feasible for large‐scale screening due to the already high demands on clinicians. To address this challenge, digital solutions could facilitate broad and efficient screening, particularly in decentralized settings.^7^, ^8^ Automatic speech analysis has emerged as a promising tool, offering a low‐cost, low‐burden alternative to traditional cognitive assessments.^9^ Speech biomarkers, such as the speech biomarker for cognition (SB‐C), ^10^ have shown potential for detecting MCI and have been validated for this purpose.^11^

Speech and language impairments are early and prominent symptoms of AD. These difficulties often manifest as word‐finding problems, particularly in naming familiar objects. Early in the disease, individuals experience increased pauses during speech and a decline in verbal fluency and lexical retrieval.^12^ These subtle changes in speech patterns can be quantified using automated speech analysis, offering a scalable and sensitive way to capture early cognitive change. Importantly, digital speech assessments do not aim to measure language impairment in isolation; rather, they use speech production as an ecologically valid behavioral channel that engages multiple cognitive processes. In this way, subtle inefficiencies in memory retrieval, executive control, and attentional regulation can manifest in naturalistic performance and be detected through standardized speech tasks.^13^ Previous studies have shown that automatic speech feature extraction during cognitive tasks, even when conducted over simple phone calls, can reliably identify individuals exhibiting early signs of cognitive decline.^14^, ^15^, ^16^ Notably, the SB‐C has demonstrated robust sensitivity in detecting early‐stage cognitive impairment across diverse cohorts and languages, achieving an area under the curve (AUC) of ≈ 0.81 for differentiating cognitively unimpaired (CU) persons from impaired.^11^ The SB‐C is derived from 70 distinct speech features extracted from cognitive tasks, such as semantic verbal fluency and word learning tests.^10^ This biomarker has undergone rigorous validation following the Digital Medicine Society's V3 framework^17^ and is currently used in multiple AD clinical trials as a pre‐screening tool for MCI detection. The assessment is fully automated and can be completed remotely via phone or app. Speech‐based digital cognitive assessments are not intended to replace classical neuropsychological testing or to diagnose specific cognitive syndromes. Instead, they aim to provide scalable, low‐burden measures of functional cognitive efficiency by quantifying behavioral manifestations of cognitive processes during structured tasks. Within this framework, speech tasks such as verbal fluency and narrative recall impose demands on lexical retrieval, attentional control, executive organization, and processing speed, allowing indirect indexing of cognitive efficiency without attempting fine‐grained linguistic diagnosis.

Despite these advancements, to implement speech‐based measures for the detection of MCI, it is crucial to validate their applicability across varied study populations and geographic regions. Ensuring this validation is essential for establishing their reliability as diagnostic support, pre‐screening criteria, or as effective tools for large‐scale cognitive assessments.

To date, most research on speech and language biomarkers in AD has focused on cross‐sectional datasets collected in single–language, controlled clinical settings. Several reviews have highlighted the field's reliance on monolingual samples and in clinic‐based assessments, limiting generalizability to real‐world or multilingual contexts.^18^, ^19^ In response, the Screening over Speech in Unselected Populations for Clinical Trials in Alzheimer's Disease (PROSPECT‐AD) project^20^ aims to derive and validate remotely collected longitudinal speech biomarkers across multiple European cohorts spanning preclinical to early AD, and here we report baseline associations among SB‐C, clinical profiles, and cerebrospinal fluid (CSF) biomarkers using remote and in‐clinic speech‐based testing.^20^

**RESEARCH IN CONTEXT**

1. **Systematic review**: We reviewed literature using PubMed and Google Scholar to identify studies examining speech and language‐based digital biomarkers in Alzheimer's disease (AD). Prior work has shown that acoustic and linguistic features can differentiate individuals with mild cognitive impairment or AD from healthy controls. However, most studies are single‐cohort, monolingual, and lack validation against biological markers of AD pathology.
2. **Interpretation**: This multi‐cohort study across European sites demonstrates that a fully automated speech‐based cognitive assessment (SB‐C) can distinguish between cognitively unimpaired and impaired individuals and is associated with cerebrospinal fluid amyloid beta and phosphorylated tau181 status. Notably, we found evidence of SB‐C score differences even in participants with subjective cognitive decline stratified by amyloid/tau biomarker profiles, highlighting early language‐related impairments linked to AD pathology.
3. **Future directions**: Our findings suggest SB‐C may support remote, low‐burden screening in decentralized clinical trials. Longitudinal analyses will explore its predictive validity for cognitive decline and disease progression. Integration with other digital tools (e.g., passive monitoring, imaging) and harmonization across biomarker platforms may enhance sensitivity and utility in early detection frameworks.

## 2 METHODS

### 2.1 Participants

This study includes participants from five observational cohorts: the Barcelonaβeta Brain Research Center's Alzheimer's at‐risk cohort (β‐AARC) study in Spain, the European Prevention of Alzheimer's Dementia (EPAD) Scotland follow‐on study in the UK, the Dementia Study of Cognitive and Biomarker Dynamics (DESCRIBE) and the Longitudinal Cognitive Impairment and Dementia Study (DELCODE) studies in Germany,^21^ and the Biomarkers for Identifying Neurodegenerative Disorders Early and Reliably (BioFINDER)‐Primary Care study (NCT06120361) in Sweden.^22^

For the present study, participants were eligible for inclusion if they were aged ≥40 years and exhibited cognitive function ranging from CU to mild dementia. The lower age threshold varied across cohorts due to their independent study designs and recruitment strategies. While some cohorts targeted older populations typical of preclinical AD research, others—particularly those recruiting from primary care—aimed to capture individuals presenting with cognitive symptoms from midlife onward, including potential early‐onset presentations. Individuals aged 40 to 55 represented a small proportion of the sample. Additional inclusion criteria included the availability of diagnosis‐specific biomarkers, fluency in the study language, and the ability to provide informed consent independently or via a legally authorized representative. All participants were required to sign and date an informed consent form prior to enrollment.

Exclusion criteria included significantly impaired hearing that could interfere with speech assessments, any significant and unstable systemic illness or organ failure that could hinder participation, and current substantial alcohol or substance misuse. Individuals who declined further investigation at the memory clinic or whose cognitive impairment could be definitively attributed to another medical or psychiatric condition (e.g., anemia, infection, severe sleep deprivation, psychotic disorder, moderate to severe depression) were also excluded from participation.

As this study was integrated into existing longitudinal cohorts, inclusion and exclusion criteria were defined independently within each cohort.

In the β‐AARC study, participants were aged 55 to 80 years and presented with either subjective cognitive decline (SCD) or MCI. Additional inclusion criteria included literacy, fluency in Spanish or Catalan, a score ≤ 45 on the Memory Alteration Test (M@T), and the availability of an informant to provide collateral information. Exclusion criteria comprised major psychiatric disorders (e.g., major depression, schizophrenia), neurological conditions (e.g., Parkinson's disease, epilepsy, multiple sclerosis), contraindications to magnetic resonance imaging (MRI) or lumbar puncture, significant brain injury, or other medical conditions that could interfere with study participation.

The EPAD Scotland follow‐up study included literate individuals aged ≥50 years with at least seven years of formal education, and literacy in the study language. A study partner was also required. Exclusion criteria included a diagnosis of dementia (Clinical Dementia Rating [CDR] ≥ 1); known autosomal dominant AD mutations; and medical, neurological, or psychiatric conditions with potential to impact cognitive performance (e.g., Parkinson's disease, Huntington's disease, major depression, substance misuse).

In the DESCRIBE and DELCODE cohorts, eligible participants were aged ≥ 50 years and presented with cognitive complaints. Inclusion required informed consent and the availability of a study partner. Participants were excluded if they had a diagnosis of dementia, significant neurological or psychiatric disorders, or contraindications to key procedures such as MRI or lumbar puncture.

The BioFINDER‐Primary Care study enrolled patients aged ≥ 40 years who consulted primary care for cognitive symptoms reported by themselves, an informant, or identified by the physician. Eligible participants were classified as having SCD, MCI, or mild dementia. Exclusion criteria included a prior diagnosis of dementia, unstable systemic illness, significant alcohol or substance misuse, refusal of memory clinic referral, cognitive impairment due to acute events such as stroke, or conditions clearly responsible for cognitive symptoms (e.g., psychosis, depression, or substance abuse), as determined by the primary care physician.

For the present analyses, participants were included only if they had complete speech data for both Semantic Verbal Fluency (SVF) and Verbal Learning Task (VLT), as well as the corresponding clinical outcome measures required for each analysis. Participants with missing SVF or VLT recordings, incomplete or unusable speech data, or missing core clinical variables (e.g., Mini‐Mental State Examination [MMSE], CDR, or biomarker status where applicable) were excluded from the respective analyses. As a result, all analyses were conducted using complete‐case datasets specific to each outcome.

### 2.2 Speech data

Speech data were collected in a fully automated way via the ki:elements’ Mili platform (a digital software node that initiates and administers the assessment), either in clinic (BioFINDER‐Primary Care) or over the phone at baseline (DESCRIBE and DELCODE, EPAD Scotland, and β‐AARC) as well as at follow‐up visits, which differed by cohort; either at month 3 (T3) and/or month 6 (T6) and month 12 (T12). A detailed study overview can be found in Table S1 in supporting information.

Specifically, after giving consent, participants were contacted by the study coordinator to schedule the appointments for the automated phone assessments. During each call, Mili asked the participants to confirm consent, reminded them that the call was recorded, and instructed them to avoid sharing identifiable information. During the automated assessment calls, Mili gave instructions verbatim, and participants confirmed understanding before starting. The assessment included the 15‐word VLT (or 10‐word VLT from the Repeatable Battery for the Assessment of Neuropsychological Status [RBANS] in the BioFINDER‐Primary Care study), SVF, and a narrative storytelling task. Upon completion in the remote administrations, Mili thanked participants for their time.

Across all cohorts, at baseline, the semantic verbal fluency task used the “animals” category with a standardized instruction in each language (“Name as many animals as you can think of in 1 minute”). The storytelling task followed a common Mili platform script across languages (Spanish, Catalan, German, English, Swedish) using a standardized prompt (e.g., “Can you tell us about a positive/negative event?”). Instructions for all administered tasks (semantic fluency, verbal learning, and storytelling) were similar and standardized using a shared script derived from the Mili platform and translated through a forward–backward workflow with language‐specific adaptation and review. Administration modality was consistent within cohorts and was predominantly phone‐based, with BioFINDER‐Primary Care using app‐based administration at baseline. Automated quality‐control procedures were applied to speech recordings (e.g., minimum signal quality thresholds and task completion checks); however, remote administration inherently limits control over factors such as environmental conditions.

The SB‐C digital assessment composite score is derived from speech recordings from two common neuropsychological assessments, namely the VLT and the SVF. Speech from both assessments underwent automated processing using the ki:elements’ proprietary speech analysis pipeline. This involved automatic speech recognition (ASR) to transcribe speech and extract features capturing various aspects of verbal output, including semantic, temporal, and task‐related features with high concordance to manual transcriptions.^23^ For the construction of the SB‐C global cognition score, 70 features were used, which also contributed to three distinct neurocognitive subdomain scores: learning and memory, executive function, and processing speed. These subdomain scores are modeled as speech‐derived indices intended to reflect domain‐relevant variation during task performance, rather than direct measures of specific cognitive faculties. Finally, these subdomain scores were combined to derive the single aggregated global cognition score.

SB‐C is derived from groups of speech features reflecting temporal organization (e.g., word transition time, pause structure), fluency dynamics (e.g., production rate, semantic clustering and switching), and retrieval efficiency (learning slopes) during structured cognitive tasks. These features are not intended to map one‐to‐one onto classical linguistic error categories (e.g., aphasic paraphasias), but rather to capture functional manifestations of cognitive load, attentional control, and retrieval efficiency during task performance. Feature selection and aggregation followed a predefined model validated for reliability and clinical relevance in prior work.^10^, ^11^

ASR models and feature extraction pipelines were optimized per language, and SB‐C scores were normalized within language groups before cross‐cohort analyses. Although the full feature‐engineering and aggregation pipeline underlying SB‐C is proprietary and cannot be disclosed in full detail, the conceptual structure of the model—including its decomposition into domain‐related scores (learning and memory, executive function, and processing speed), the underlying feature categories, and the validation framework—has been described in prior publications.^10^, ^11^, ^49^

Additionally, for each participant in the study, demographic and clinical information was collected, during their regular cohort visit including biomaterial for research purposes such as CSF biomarkers.

### 2.3 Clinical diagnosis

Disease severity was assessed using the CDR scale and a detailed neuropsychological assessment was administered to comprehensively evaluate cognitive function and disease progression. Detailed descriptions of cohort‐specific clinical assessments and biomarker collection procedures are reported in the PROSPECT‐AD protocol publication.^20^

In the β‐AARC study clinical diagnosis was made after collection of baseline data, including a clinical assessment by a trained neurologist, cognitive and lifestyle questionnaires, blood extraction, MRI scanning, and an extensive cognitive and functional assessment conducted by a neuropsychologist. Objective impairment in cognitive performance was considered for scores below –1.5 standard deviation (SD), using age‐ and education‐adjusted healthy population‐derived reference norms. Uncertain cases underwent review at multidisciplinary clinical meetings and final diagnostic labels were assigned. SCD was defined following^24^ guidelines as subjective experience of cognitive decline intense enough to seek medical help in absence of cognitive or functional impairments compatible with MCI or dementia. MCI was defined according to National Institute on Aging–Alzheimer's Association^25^ criteria.

In the EPAD Scotland follow‐up study, participants were considered to have a diagnosis if they self‐reported a prior clinical diagnosis of MCI or early AD dementia by a physician, in accordance with standard diagnostic criteria (e.g., National Institute on Neurological and Communicative Disorders and Stroke–Alzheimer's Disease and Related Disorders Association [NINCDS/ADRDA] or Diagnostic and Statistical Manual of Mental Disorders Fifth Edition [DSM‐5]); a CDR score > 0.5 was used solely as an exclusion criterion for new participants at baseline.

In the DESCRIBE cohort, diagnosis was based on a review process focusing on neuropsychological and clinical observations and not necessarily on biomaterial. Specifically, in the DELCODE study, patients were classified as SCD when upon evaluation they reported cognitive decline causing concern and sought evaluation at the memory clinic within the last 6 months to 5 years. They also scored within the normal range (1.5 SD) on all Consortium to Establish a Registry for Alzheimer's Disease (CERAD) tests during their diagnostic evaluation. For MCI, patients had to score < −1.5 SD below the normal range in the delayed recall of the CERAD word list; cognitive decline was reported by the patient, an informant, or the treating physician; and criteria for dementia were not met. In the DESCRIBE study, MCI (including amnestic and non‐amnestic MCI) was classified based on the Winblad/Petersen criteria.^26^ For both cohorts the NINCDS/ADRDA criteria were used for AD diagnosis.^27^

In BioFINDER‐Primary Care, SCD was defined as experiencing cognitive symptoms to the level that led the patient to seek help in primary care but not fulfilling the criteria for MCI or dementia. MCI was diagnosed in weekly consensus rounds including the responsible dementia specialist and neuropsychologist, based on the presence of significant cognitive symptoms and abnormal cognitive test results using the RBANS battery (accounting for premorbid cognitive level). The MCI definition did not require that a strict threshold in a cognitive domain was met (although all performed <−1 SD in at least one cognitive domain in the RBANS battery) but was based on the overall clinical assessment. The classification followed the design of the MCI classification of the Mayo Clinic Study of Aging^28^ and was in line with the DSM‐5 criteria for mild neurocognitive disorder.^29^ Dementia was diagnosed according to the DSM‐5 criteria for major neurocognitive disorder.^29^

Diagnostic labels (CU/SCD/MCI/dementia) were assigned within each cohort according to that cohort's original study protocol and therefore differed across studies. Because cross‐cohort harmonization of diagnostic definitions was not feasible, a cohort‐by‐cohort overview of key cognitive assessments and diagnostic approaches is provided in Table S2 in supporting information.

### 2.4 CSF procedures

In the β‐AARC study, CSF samples were obtained by lumbar puncture following standard procedures.^30^ Amyloid beta (Aβ)42 and phosphorylated tau (p‐tau)181 were measured using Lumipulse assays and local cut‐offs were applied: A+ was considered for Aβ42 levels <  750 pg/mL, and T+ for p‐tau181 >  69.85 pg/mL.^31^ For AT status, A was defined as Aβ42 <  750 pg/mL and T as p‐tau181 >  69.85 pg/mL in β‐AARC.

In the EPAD Scotland follow‐up study, CSF was used from the last available time point in the EPAD Longitudinal Cohort Study (LCS). CSF was collected in the EPAD LCS according to local protocols and measured for Aβ42, total tau (t‐tau), and p‐tau181 in a single laboratory at the Clinical Neurochemistry Laboratory at the University of Gothenburg, Sweden, using the Roche cobas Elecsys System. For AT status, A was defined as Aβ42 <  1000 pg/mL and T as p‐tau181 >  27 pg/mL in EPAD Scotland.

In the DESCRIBE cohort, CSF samples were collected during routine diagnostic work‐up in participating memory clinics, following standard clinical procedures. Core AD biomarkers (Aβ42, t‐tau, and p‐tau181) were analyzed using validated immunoassays (e.g., INNOTEST). In the DELCODE study, CSF collection was optional and conducted at baseline. Biomarker analyses were centralized and performed using standardized enzyme‐linked immunosorbent assay methods. A+ and T+ classifications were based on internally validated thresholds, consistent with AD biomarker research guidelines. For AT status, A was defined as Aβ42 <  638.7 pg/mL and T as p‐tau181 >  73.65 pg/mL in DESCRIBE/DELCODE AD.

In the BioFINDER‐Primary Care study, CSF was collected according to a standardized protocol.^22^ Aβ42, Aβ40, and p‐tau181 were analyzed using the US Food and Drug Administration–approved Lumipulse assays. For AT status, A was defined as Aβ42/Aβ40 <  0.072 pg/mL and T as p‐tau181/Aβ40 >  0.046 pg/mL in BioFINDER‐Primary Care.

### 2.5 Outcomes

Across all cohorts, key clinical and cognitive outcome measures were used to evaluate the validity and clinical relevance of the SB‐C digital cognition scores. Clinical staging was assessed using the CDR scale, which served as a core indicator of disease severity. In the BioFINDER‐Primary Care study, the CDR was scored prospectively by the treating physician, while in DESCRIBE, DELCODE, and β‐AARC, it was assigned through a clinical consensus process. In the EPAD follow‐up study, a CDR score > 0.5 served as an exclusion criterion for new participants at baseline. Global cognitive performance was further evaluated using the MMSE, which was administered across all cohorts except EPAD Scotland. The MMSE served both as a screening tool and as a continuous measure of cognitive impairment, enabling comparison to SB‐C scores.

In addition, composite cognitive outcomes were used to capture early cognitive changes, particularly in at‐risk individuals. The Preclinical Alzheimer Cognitive Composite (PACC) or modified versions thereof were available in DELCODE and DESCRIBE, combining performance across memory, executive function, and global cognition domains. These composite scores were used to test the convergent validity of the SB‐C subdomain and global scores.

Cohort‐specific cognitive assessments were also included. In BioFINDER‐Primary Care, cognitive function was measured using the RBANS, which covers multiple domains including immediate and delayed memory, visuospatial skills, and language. In DELCODE, the CERAD neuropsychological battery was administered and used both for diagnostic classification and longitudinal follow‐up, serving as a standard reference for memory‐related cognitive impairment.

Where available, clinical diagnosis or diagnostic conversion over time (e.g., from SCD to MCI or from MCI to dementia) was also used as an outcome to evaluate the predictive utility of SB‐C scores. Diagnostic conversion was tracked over a 12‐month follow‐up period in DELCODE, β‐AARC, and BioFINDER‐Primary Care. Collectively, these outcome measures enabled a comprehensive assessment of the SB‐C scores in relation to standard clinical and cognitive benchmarks across multiple independent cohorts.

### 2.6 Statistical analysis

Spearman rank correlations were calculated between the SB‐C cognition score and clinical measures (MMSE total score and CDR global score). To compare cognitively healthy individuals (healthy controls or SCD) and cognitively impaired individuals (MCI or mild dementia), inferential statistics were conducted using a non‐parametric Kruskal–Wallis test due to non‐normal data distribution and/or unequal variances.

Group comparisons across cognitive stages were conducted using analyses of covariance (ANCOVA). SB‐C scores were tested for their ability to significantly differentiate the different cohorts between CU, including healthy controls (HCs) and SCD, versus cognitively impaired (CI), including MCI and mild dementia (*P* < 0.001), while controlling for age, education, and sex. Comparisons of SB‐C subdomains (executive function, memory, processing speed) were also conducted using ANCOVA and found to vary significantly across groups, reinforcing the SB‐C's sensitivity in distinguishing different levels of cognitive impairment. No imputation was performed for missing data; all statistical analyses were conducted using complete cases only.

Additionally, optimal cut‐offs for the SB‐C for discriminating between CSF Aβ positive versus negative and CSF p‐tau181 positive (true positive [TP], false positive [FP]) versus negative (true negative [TN], false negative [FN]) participants were determined by maximizing the risk ratio (TP / [TP + FN]) / (FP / [FP + TN]) for Aβ, and the F1 score (2 * TP) / (2 * TP + FP + FN) for p‐tau. For Aβ positivity, a cut‐off of 0.4622 was established based on the EPAD Scotland cohort and subsequently applied separately to the β‐AARC, EPAD Scotland, and BioFINDER‐Primary Care cohorts. For p‐tau positivity, a cut‐off of 0.4374 was derived from the combined EPAD and β‐AARC and then applied to each cohort individually. Sensitivity, specificity, balanced accuracy, and the area under the receiver operating characteristic curve (ROC AUC) were reported as performance metrics to validate these cut‐offs.

Performance metrics including sensitivity, specificity, balanced accuracy, F1 score, ROC AUC, and the Matthews Correlation Coefficient (MCC) were calculated from the resulting confusion matrices. Ninety‐five percent confidence intervals for all metrics were estimated using bootstrap resampling with 1000 iterations to account for sampling variability. The analysis assumes independent observations and correctly labeled outcomes. In addition, the MCC was reported as a balanced summary metric of classification performance. MCC integrates true positives, true negatives, false positives, and false negatives into a single measure ranging from −1 (complete disagreement) to +1 (perfect prediction), with 0 indicating performance no better than chance.[Table alz71462-tbl-0001]

To evaluate the association between speech‐based cognitive scores and AD biomarker profiles, participants were grouped according to their AT status based on CSF Aβ42 and p‐tau181 levels: A–T–, A+T–, and A+T+. The A–T+ group was not included in formal comparisons due to its low prevalence and heterogeneous clinical significance. SB‐C cognition scores were compared across AT groups within each cohort (β‐AARC, EPAD Scotland, and BioFINDER‐Primary Care) using Kruskal–Wallis tests. This non‐parametric test was chosen due to non‐normal score distributions and unequal group sizes. Statistical significance was set at *P* < 0.05. Adjustment for additional clinical or genetic covariates was not feasible due to inconsistent availability across cohorts.

## 3 RESULTS

### 3.1 Participant characteristics

A total of 736 participants (mean [SD] age, 71.37 [7.71] years; 459 females [62.7%], 277 males [37.3%]) were included in the study. The β‐AARC cohort included 85 participants (mean [SD] age, 65.89 [6.82] years; 52 females [61.2%]). The EPAD Scotland cohort consisted of 77 participants (mean [SD] age, 70.18 [6.48] years; 42 females [54.5%]). The DESCRIBE and DELCODE cohorts included 159 participants (mean [SD] age, 72.82 [7.20] years; 75 females [47.2%]), and the BioFINDER‐Primary Care cohort included 415 participants (mean age 76.16 [7.5] years; 290 females [69.9%]). Table 1 presents the detailed cross‐sectional characteristics of the cohort participants. Across all cohorts, education levels varied, with the highest mean years of education observed in the EPAD Scotland cohort (16.38 [3.86] years) and the lowest in the BioFINDER‐Primary Care cohort (10.48 [2.98] years). Cognitive function, as measured by MMSE, was highest in the DESCRIBE and DELCODE cohorts (mean: 29.41 [0.97]) and lowest in the BioFINDER‐Primary Care cohort MCI and dementia groups (mean: 25.61 [5.08] and 23.07 [4.21], respectively). The SB‐C scores progressively decreased with increasing severity of cognitive impairment, with the highest mean scores observed in HCs (EPAD Scotland, mean  =  0.71, SD  =  0.18) and the lowest in individuals with dementia (BioFINDER‐Primary Care, mean  =  0.17, SD  =  0.16).

**TABLE 1 alz71462-tbl-0001:** Cross‐sectional characteristics of cohort participants. Mean (SD) are reported for the variables age, education, MMSE, and ki:E SB‐C. *N* (%) are reported for categorical variables.

	PROSPECT‐AD						
Study	β‐AARC	EPAD Scotland	DESCRIBE/DELCODE AD		BioFINDER‐Primary Care		
Group	SCD	HC	HC/ SCD	MCI	SCD	MCI	Dementia
N	85 (52 F)	77 (42 F)	111 (50 F)	48 (25 F)	132	169	114
Age	65.89 (6.82)	70.18 (6.48)	79.72 (7.15)	72.88 (7.38)	72.79 (7.67)	77.14 (6.91)	78.92 (6.54)
Education	15.74 (3.53)	16.38 (3.86)	15.19 (2.76)	14.46 (2.62)	12.17 (3.24)	10.60 (2.72)	10.53 (2.95)
MMSE	28.96 (1.29)	–	29.41 (0.97)	27.81 (2.00)	26.77 (6.61) NA: 1	25.61 (5.08)	23.07 (4.21) NA: 1
CDR Global score	‐						
0		67	70	11	123	24	1
0.5		10	28	33	1	133	54
1							40
2							12
Missing/NA					8	12	7
ki:e SB‐C	0.58 (0.18)	0.53 (0.19)	0.71 (0.18)	0.46 (0.25)	0.51 (0.14)	0.33 (0.14)	0.17 (0.16)
AT status, no. (%)							
A–T–	32 (54.24)	25 (3.19)		33 (67.35)	69 (52.3)	54 (32.0)	23 (20.2)
A+T–	20 (33.90)	13 (27.66)		11 (22.45)	23 (17.4)	13 (7.7%)	6 (5.3%)
A+T+	3 (5.08)	7 (14.89)		2 (4.08)	16 (18.2)	58 (34.3%)	61 (53.5%)
A–T+	4 (6.78)	2 (4.26)		3 (6.12)		Not used	Not used
						NA: 41 (24.3)	NA: 19 (16.7)

*Notes*: For AT status, A was defined as Aβ42 < 750 pg/mL and T as p‐tau181 > 69.85 pg/mL in β‐AARC. For AT status, A was defined as Aβ42< 1000 pg/mL and T as p‐tau181 > 27 pg/mL in EPAD Scotland. For AT status, A was defined as Aβ42< 638.7 pg/mL and T as p‐tau181 > 73.65 pg/mL in DESCRIBE/DELCODE AD. For AT status, A was defined as Aβ42/Aβ40 <  0.072 pg/mL and T as p‐tau181/Aβ40 >  0.046 pg/mL in BioFINDER‐Primary Care.

Abbreviations: Aβ, amyloid beta; β‐AARC, Barcelonaβeta Brain Research Center's Alzheimer's at‐risk cohort; BioFINDER, Biomarkers for Identifying Neurodegenerative Disorders Early and Reliably; CDR, Clinical Dementia Rating; DELCODE, Longitudinal Cognitive Impairment and Dementia Study; DESCRIBE, Dementia Study of Cognitive and Biomarker Dynamics; EPAD, European Prevention of Alzheimer's Dementia; HC, healthy control; MCI, mild cognitive impairment; MMSE, Mini‐Mental State Examination; NA, not available; SB‐C, speech biomarker for cognition; SCD, subjective cognitive decline; SD, standard deviation.

### 3.2 Comparisons to gold standard measures

**FIGURE 1:**
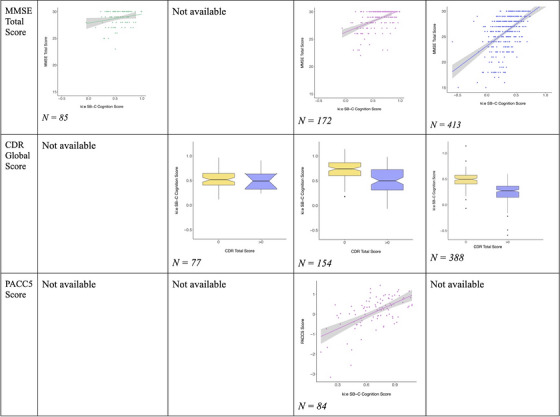
Comparisons to clinical gold standard measures. CDR, Clinical Dementia Rating; DELCODE, Longitudinal Cognitive Impairment and Dementia Study; MMSE, Mini‐Mental State Examination; PACC, Preclinical Alzheimer Cognitive Composite; SB‐C, speech biomarker for cognition.

Spearman correlations were conducted to assess the associations between SB‐C cognition scores and standard clinical assessments across multiple cohorts (Figure 1) and Kruskal–Wallis tests were used to differentiate between CDR Global score groups.

In the DESCRIBE and DELCODE cohorts, SB‐C cognition scores demonstrated a positive correlation with the MMSE (*r* = 0.44, *P* < 0.001) and the PACC‐5 score (*r* = 0.51, *P* < 0.001). These results indicate that higher SB‐C scores were associated with better cognitive function. In the BioFINDER‐Primary Care cohort, SB‐C scores correlated with MMSE (*r* = 0.49, *P* < 0.001) and were significantly different between CDR groups (*P* < 0.001), in the same directions as for DESCRIBE/DELCODE, further supporting the validity of SB‐C as a measure of cognitive status. In contrast, the EPAD Scotland cohort did not show significant group differences between CDR groups. The β‐AARC study revealed a weak correlation between MMSE and SB‐C executive function (*r* = 0.23, *P* < 0.05), whereas associations with the other SB‐C measures were non‐significant. Detailed correlation coefficients are presented in Table 2.

**TABLE 2:** Correlations/group comparison of speech biomarker with gold standard clinical measures. For MMSE and PACC‐5, correlations are reported as coefficient (*P* value). For CDR, (*P* value) of Kruskal–Wallis test is reported.

		SB‐C scores			
Anchor	Cohort	Cognition score	Executive function	Memory	Processing speed
**MMSE**	**DESCRIBE / DELCODE**	0.44 (<0.001)	0.41 (<0.001)	0.35 (<0.001)	0.41 (<0.001)
	**β‐AARC**	0.15 (0.15)	0.23 (<0.05)	0.12 (0.27)	0.07 (0.49)
	**BioFINDER‐ Primary Care**	0.49 (<0.001)	0.34 (<0.001)	0.49 (<0.001)	0.47 (<0.001)
**CDR**	**DESCRIBE / DELCODE**	(<0.001)	(<0.001)	(<0.001)	(<0.001)
	**EPAD Scotland**	(0.65)	(0.60)	(0.77)	(0.77)
	**BioFINDER‐Primary Care**	(<0.001)	(<0.001)	(<0.001)	(<0.001)
**PACC‐5**	**DESCRIBE / DELCODE**	0.51 (<0.001)	0.57 (<0.001)	0.33 (<0.001)	0.38 (<0.001)

Abbreviations: β‐AARC, Barcelonaβeta Brain Research Center's Alzheimer's at‐risk cohort; BioFINDER, Biomarkers for Identifying Neurodegenerative Disorders Early and Reliably; CDR, Clinical Dementia Rating; DELCODE, Longitudinal Cognitive Impairment and Dementia Study; DESCRIBE, Dementia Study of Cognitive and Biomarker Dynamics; EPAD, European Prevention of Alzheimer's Dementia; MMSE, Mini‐Mental State Examination; PACC, Preclinical Alzheimer Cognitive Composite; SB‐C, speech biomarker for cognition; SCD, subjective cognitive decline.

### 3.3 Group comparisons across cognitive stages

**FIGURE 2:**
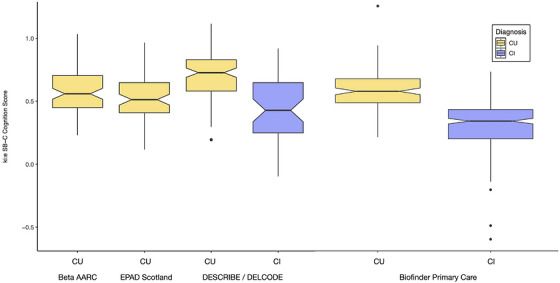
SB‐C scores across cohorts (Beta AARC *N* = 85, EPAD Scotland *N* = 77, DESCRIBE/DELCODE *N* = 159, Biofinder Primary Care *N* = 415). Beta AARC, Barcelonaβeta Brain Research Center's Alzheimer's at‐risk cohort; Biofinder, Biomarkers for Identifying Neurodegenerative Disorders Early and Reliably; CI, cognitively impaired; CU, cognitively unimpaired; DELCODE, Longitudinal Cognitive Impairment and Dementia Study; DESCRIBE, Dementia Study of Cognitive and Biomarker Dynamics; EPAD, European Prevention of Alzheimer's Dementia; SB‐C, speech biomarker for cognition.

Using ANCOVA, SB‐C scores were found to significantly differentiate between CU, including HC and SCD, against CI, including MCI and mild dementia (*P* < 0.001), controlling for age, education, and sex (see Figure 2). SB‐C subdomain scores also differed across groups; these values are reported as modeled speech‐derived indices and should be interpreted cautiously (see section 4). In addition, the SB‐C cognition score demonstrated to have good classification performance for distinguishing CI from CU participants, with ROC AUCs of 0.70 in the DESCRIBE and DELCODE cohorts and 0.82 in the BioFINDER‐Primary Care cohort, indicating strong discriminative ability across clinical settings.

### 3.4 Biomarker classification and reference cutoffs

The SB‐C cognition score was further analyzed for its ability to classify CSF Aβ and p‐tau181 biomarker positivity only in participants with SCD and MCI (see Tables 3 and 4). Across cohorts, the accuracy for Aβ classification ranged from 0.63 in the EPAD Scotland cohort to 0.69 in the BioFINDER‐Primary Care cohort. AUC values were highest in the BioFINDER‐Primary Care cohort (0.74) and lowest in EPAD (0.56). For p‐tau181 classification, balanced accuracy was highest in the BioFINDER‐Primary Care cohort (0.73) and lowest in EPAD (0.63), while AUC values ranged from 0.72 in EPAD to 0.82 in BioFINDER‐Primary Care. Calibration plots are provided in Figure S1 in supporting information and showed variable agreement between predicted and observed event rates across cohorts, with less stable calibration at higher predicted probabilities. The classification results indicate that SB‐C scores can differentiate biomarker‐positive individuals, with the strongest performance observed in the BioFINDER‐Primary Care cohort. Across cohorts, MCC values ranged from 0.22 to 0.44, indicating modest overall agreement between SB‐C classifications and biomarker status (see detailed results in Table S3 in supporting information). In the DESCRIBE and DELCODE cohorts, CSF data were, on average, collected 4 to 5 years prior to the speech assessment, limiting the temporal alignment between biomarker and speech measures. As a result, these data were not included in the comparisons to the SB‐C measures. Table 3 provides a detailed summary of these classification results.

**TABLE 3:** Classification of Aβ group results in each cohort.

Cohort	SB‐C score cut‐off	Balanced accuracy	ROC AUC	AUC 95% CI	Sensitivity	Specificity
β‐AARC	0.4622	0.64	0.69	0.54–0.83	0.50 (0.29–0.71)	0.77 (0.62–0.90)
EPAD Scotland	0.4622	0.63	0.56	0.34–0.74	0.60 (0.33–0.83)	0.66 (0.49–0.82)
BioFINDER‐ Primary Care	0.4622	0.69	0.74	0.67–0.81	0.74 (0.72–0.78)	0.64 (0.52–0.74)

Abbreviations: Aβ, amyloid beta; AUC, area under the curve; β‐AARC, Barcelonaβeta Brain Research Center's Alzheimer's at‐risk cohort; BioFINDER, Biomarkers for Identifying Neurodegenerative Disorders Early and Reliably; CI, confidence interval; EPAD, European Prevention of Alzheimer's Dementia; ROC, receiver operating characteristic; SB‐C, speech biomarker for cognition.

**TABLE 4:** Classification of p‐tau groups results in each cohort.

Cohort	SB‐C score cut‐off	Balanced accuracy	ROC AUC	AUC 95% CI	Sensitivity	Specificity
β‐AARC	0.4374	0.71	0.82	0.70–0.94	0.71 (0.33–1.00)	0.71 (0.57–0.83)
EPAD Scotland	0.4374	0.63	0.72	0.56–0.88	0.56 (0.22–0.88)	0.71 (0.56–0.85)
BioFINDER‐ Primary Care	0.4374	0.73	0.82	0.77–0.88	0.77 (0.71–0.79)	0.70 (0.58–0.77)

Abbreviations: AUC, area under the curve; β‐AARC, Barcelonaβeta Brain Research Center's Alzheimer's at‐risk cohort; BioFINDER, Biomarkers for Identifying Neurodegenerative Disorders Early and Reliably; CI, confidence interval; EPAD, European Prevention of Alzheimer's Dementia; p‐tau, phosphorylated tau; ROC, receiver operating characteristic; SB‐C, speech biomarker for cognition.

### 3.5 Group comparisons by AT status

**FIGURE 3:**
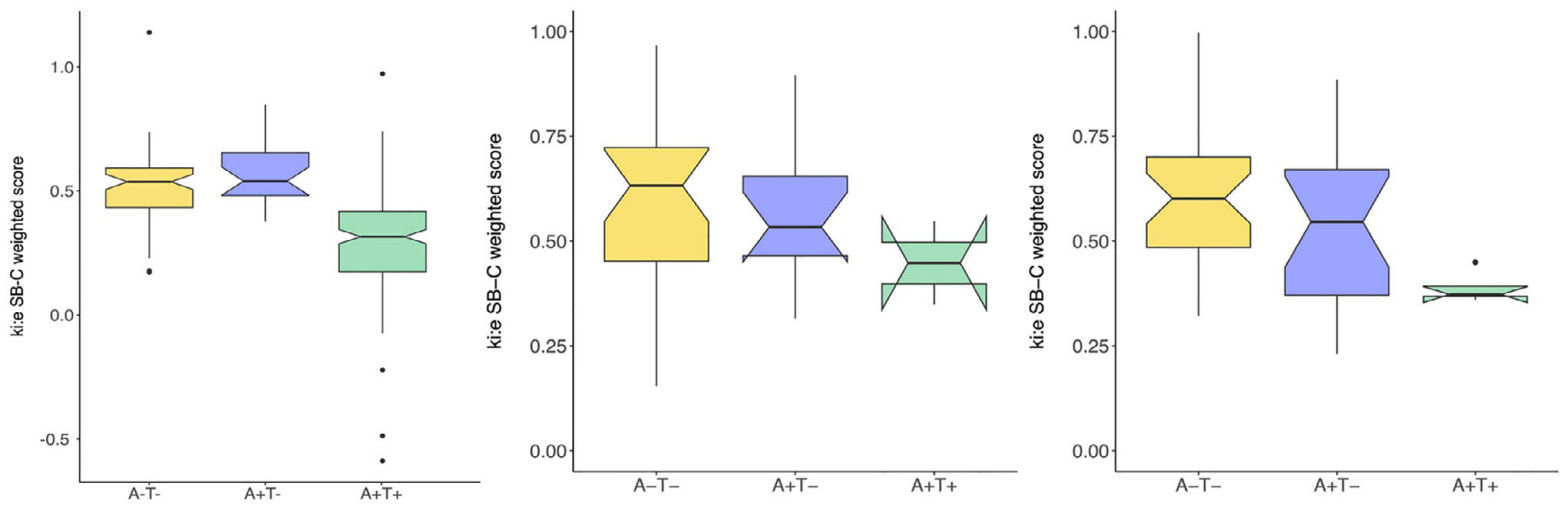
Group comparisons of SB‐C across AT status (from left to right: β‐AARC, EPAD Scotland, BioFINDER‐Primary care). χ^2^ and *P* value of Kruskal–Wallis test are reported. A, amyloid; β‐AARC, Barcelonaβeta Brain Research Center's Alzheimer's at‐risk cohort; BioFINDER, Biomarkers for Identifying Neurodegenerative Disorders Early and Reliably; EPAD, European Prevention of Alzheimer's Dementia; SB‐C, speech biomarker for cognition; T, tau.

Figure 3 presents group comparisons of SB‐C cognition scores across AT biomarker profiles (A–T–, A+T–, and A+T+ for three cohorts: β‐AARC, EPAD Scotland, and BioFINDER‐Primary Care. A Kruskal–Wallis test revealed a statistically significant difference in SB‐C scores across AT groups in the β‐AARC cohort (χ^2^ = 9.08, *P* < 0.05), suggesting a relationship between speech‐based cognitive performance and AD pathology in this sample. In contrast, no significant group differences were observed in the EPAD Scotland cohort (χ^2^ = 2.64, *P* = 0.27). A significant difference in SB‐C scores were found between A–T– and A+T+ groups in the BioFINDER‐Primary Care cohort (χ^2^ = 71.93, *P* < 0.001). Across cohorts, SB‐C scores were consistently lowest in individuals with the A+T+ profile and highest in A–T– individuals.

## 4 DISCUSSION

In this study, we present baseline findings from PROSPECT‐AD across five independent European cohorts, demonstrating that SB‐C captures clinically meaningful differences in cognitive status and AD pathology. SB‐C scores were associated with established cognitive measures (MMSE, CDR) and differentiated CU from CI individuals, supporting its validity as a screening‐oriented digital cognitive assessment. Associations were weaker in preclinical cohorts, likely reflecting ceiling effects and limited cognitive variability, which are well‐recognized challenges in early‐stage populations.^32^, ^33^ Nevertheless, group comparisons based on CDR ratings yielded more robust discrimination, underscoring SB‐C's sensitivity in distinguishing early cognitive impairment.

Furthermore, the associations of SB‐C scores with CSF Aβ and p‐tau181 biomarker status underscores its potential to discriminate between AD pathology positive and negative individuals. Our findings demonstrate that SB‐C scores exhibited moderate to strong classification accuracy for Aβ and p‐tau181 positivity, with the highest ROC AUC observed in the BioFINDER‐Primary Care cohort (Aβ status: 0.74, p‐tau181 status: 0.82). This suggests that speech‐based cognitive assessments might be sensitive to underlying pathological changes and useful for identifying at‐risk populations for clinical trials or early intervention.

Our findings are consistent with other studies on the use of acoustic speech parameters for predicting Aβ status, which reported an accuracy of 75% and an AUC of 0.79—outperforming conventional neuropsychological tests (AUC = 0.66).^34^ Additionally, a study assessing the capability of a fully automated speech‐based artificial intelligence system demonstrated that it could effectively detect both cognitive impairment and Aβ positivity. Speech‐based screening predicted Aβ positivity with an AUC of 0.77 and identified individuals with MCI or mild AD with an AUC of 0.83.^35^

The association between SB‐C scores and Aβ ± and p‐tau181 ± status further supports the hypothesis that language‐related cognitive impairments may manifest in early stages of AD pathology. Particularly, the significant differentiation observed in CU‐SCD individuals from the β‐AARC cohort highlights a notable and uncommon finding—namely, the ability to detect differences in speech‐based cognition by AT status within a limited sample of CU individuals. This highlights the relevance of detecting AD pathology already at the SCD stage. The relatively lower classification accuracy in other cohorts, such as EPAD, may reflect narrower cognitive variance or methodological differences. Future work should explore combining SB‐C with other sensitive digital biomarkers—such as passive monitoring or neuroimaging data—to improve classification performance in these early stages.

Recent work has linked speech features such as increased pausing and slower speech rate to tau pathology on positron emission tomography (PET) imaging, supporting the sensitivity of speech‐based measures to early AD‐related changes.^36^ While our findings validate SB‐C for distinguishing cognitive impairment stages, incorporating additional connected speech tasks might improve sensitivity to more subtle, early‐stage changes in AD pathology and will be explored in the future.

As DMTs become increasingly available, early identification of cognitive decline is critical. SB‐C may support trial recruitment and outcome assessment by offering a low‐burden, automated measure associated with CDR and composite cognitive outcomes (PACC‐5), particularly in decentralized or resource‐limited settings.

The present study has several limitations that should be considered. Cross‐linguistic differences in language structure, fluency norms, and phonemic complexity may have contributed to variability in speech‐based measures; however, analyses focused on within‐language and within‐cohort associations, and SB‐C was derived using language‐specific processing pipelines.

Although differences in predictive accuracy across cohorts may be partially driven by population characteristics, formal statistical comparisons of baseline demographic and clinical variables across all cohorts were not feasible. Access to individual‐level data was limited to the cohort‐specific variables reported in the summary tables, and harmonized datasets containing complete demographic, cognitive, and clinical information across cohorts were not available. As a result, systematic univariate or multivariate comparisons across cohorts could not be performed.

Differences in diagnostic frameworks and classification procedures including CSF biomarker assessment protocols such as the use of different p‐tau181 assays, cut‐off thresholds, and analytic laboratories likely contributed to variability in classification performance across studies. Importantly, the primary aim of this study was to evaluate the validity and predictive utility of SB‐C within each cohort, rather than to directly compare cohorts statistically, given their independent study designs, recruitment strategies, and diagnostic frameworks.

Because analyses were restricted to participants with complete speech and clinical data, the present findings may be subject to selection bias. Individuals with incomplete recordings or missing clinical information—potentially reflecting greater disease severity, technical difficulties, or reduced engagement—were excluded. This complete‐case approach may limit generalizability. Calibration was not consistently adequate across models, indicating that predicted absolute risks should be interpreted with caution and that the present findings should not be viewed as evidence of a clinically deployable risk prediction tool.

Several potentially relevant confounders (e.g., medication use, psychiatric comorbidity, apolipoprotein E genotype, and respiratory/voice‐related conditions) were not included because they were not consistently available or harmonized across cohorts. In addition, the study did not include formal negative control outcomes, as analyses focused on predefined cognitive and AD—relevant clinical and biomarker endpoints. Although cohort‐specific exclusion criteria reduce the likelihood of major confounding, residual confounding cannot be excluded and may have contributed to between‐cohort variability in SB‐C performance.

Although this study focused on middle‐aged and older adults, evidence indicates that dementia risk begins earlier in life, underscoring the need to evaluate younger populations.^37^, ^38^ The age ranges in the present study reflect the original cohort designs, and future research should evaluate speech‐based cognitive markers in younger populations to assess their potential role within early prevention frameworks.

We obtained relatively good results, particularly in the BioFINDER‐Primary Care cohort, which was the only one that started with the app‐based cognitive speech test conducted in the clinic with clinician support. This more standardized testing environment may have contributed to improved outcome results. Furthermore, clearer distinctions in SB‐C scores were observed across AT biomarker profiles in the BioFINDER‐Primary Care cohort, compared to other cohorts—supporting the sensitivity of speech‐based assessments in detecting underlying AD pathology, especially when both clinical setting and testing modality are optimized.

Remote, unassisted administration represents a trade‐off between scalability and experimental control. Unlike clinician‐administered neuropsychological assessment, remote testing limits the ability to monitor arousal, comprehension, motivation, sensory function, and compliance, and may introduce additional variance related to environmental conditions or digital familiarity. Accordingly, SB‐C should not be interpreted as a replacement for supervised neuropsychological testing, but rather as a complementary, low‐burden tool for screening and longitudinal monitoring in settings in which traditional assessment is impractical.

Future research will leverage the longitudinal data collected in the PROSPECT‐AD study to assess the predictive value of SB‐C in monitoring cognitive decline over time. By examining how SB‐C scores evolve in relation to established clinical and biomarker‐based indicators of disease progression, we aim to determine its utility not only for cross‐sectional classification but also for forecasting cognitive deterioration. This will provide critical insights into whether SB‐C can serve as a reliable marker for disease progression. Notably, prior research has already demonstrated promising outcomes in this regard, with composite speech‐based markers capturing both acoustic and linguistic alterations effectively characterizing longitudinal cognitive changes in early AD.^39^

More broadly, recent work has highlighted a shift in dementia outcome paradigms toward multidimensional and scalable measures that extend beyond traditional paper‐based cognitive tests. Recent reviews and consensus efforts emphasize the growing role of digital cognitive assessments, remote outcome measures, and biomarker‐informed frameworks for improving early detection, risk stratification, and trial efficiency.^40^, ^41^, ^42^, ^43^ In parallel, scoping and psychometric reviews have underscored both the promise and the methodological requirements of remote and unsupervised digital cognitive tools, highlighting the need for standardized validation and reporting frameworks.^33^; ^44^, ^45^ Empirical studies further demonstrate the feasibility of digital cognitive outcomes for detecting dementia and staging cognitive impairment in real‐world and primary‐care settings.^46^, ^47^, ^48^ Within this evolving landscape, speech‐based digital markers are increasingly recognized as complementary outcomes that capture subtle cognitive and functional changes while enabling low‐burden, decentralized assessment. The present findings align with these developments by demonstrating the utility of SB‐C across multiple independent cohorts and clinical contexts.

While SB‐C shows consistent associations with established cognitive measures and AD biomarkers, these associations should not be interpreted as evidence that individual speech parameters directly measure specific cognitive faculties in the classical neuropsychological sense. Rather, SB‐C captures functional speech behavior during cognitively demanding tasks, providing an indirect index of cognitive efficiency. Evidence supporting the construct validity of SB‐C comes from prior validation work demonstrating significant associations between SB‐C scores and established neuropsychological measures of global cognition and disease staging, including MMSE, CDR, and PACC‐5, as well as associations between domain‐related SB‐C composites and corresponding neuropsychological tests.^49^ As such, SB‐C should be viewed as complementary to, rather than a substitute for, detailed neuropsychological assessment, particularly in contexts requiring scalable screening or longitudinal monitoring. SB‐C is not a diagnostic tool and should not be used in isolation to establish clinical, syndromic, or pathological diagnoses, including in resource‐limited settings.

In conclusion, growing efforts are being directed toward the development of digital biomarkers, including speech‐based assessments, to enable earlier detection of cognitive decline. These approaches hold promise for enhancing precision medicine in neurodegenerative diseases by offering objective, scalable, and accessible assessment tools. However, challenges persist in ensuring their effective integration into clinical settings. The performance of speech‐based biomarkers in controlled research environments does not always seamlessly translate to real‐world clinical applications due to patient heterogeneity. Standardization initiatives will be critical for optimizing their use in primary and secondary care, in parallel with established biomarkers such as CSF and PET imaging or even blood‐based biomarkers. Moreover, issues related to accessibility, affordability, and ethical considerations—including data privacy, result disclosure, and regulatory approval—must be continuously, carefully addressed to facilitate broader adoption.

Despite these challenges, speech‐based digital biomarkers represent a significant advancement in the assessment of AD. Their ability to detect disease presence, align with disease severity, and track cognitive changes over time makes them a promising tool for both research and clinical applications.

## CONFLICT OF INTEREST STATEMENT

A.K., N.L., E.M., and J.T. are employed by the company ki:elements. Within the last 3 years S.T. has served on national and international advisory boards for Roche, Eisai, Biogen, and Grifols. He was member of the independent data safety and monitoring board of the study ENVISION (sponsor: Biogen). O.H. is an employee of Eli Lilly and Lund University, and he has previously acquired research support (for the institution) from ADx, AVID Radiopharmaceuticals, Biogen, Eli Lilly, Eisai, Fujirebio, GE Healthcare, Pfizer, and Roche. In the past 2 years, he has received consultancy/speaker fees from AC Immune, Amylyx, Alzpath, BioArctic, Biogen, Cerveau, Fujirebio, Genentech, Novartis, Roche, and Siemens. S.P. has acquired research support (for the institution) from Avid and ki elements through ADDF. In the past 2 years, he has received consultancy/speaker fees from Bioartic, Biogen, Eisai, Eli Lilly, Novo Nordisk, and Roche. S.K., P.T., G.S.B., and A.W. have nothing to disclose. Author disclosures are available in the supporting information.

## ETHICS APPROVAL

This study was approved by the respective institutional and national research ethics committees for each contributing cohort. All procedures involving human participants were conducted in accordance with the ethical standards of the relevant institutional and national research committees and with the 1964 Declaration of Helsinki and its later amendments or comparable ethical standards. This study included diverse cohorts across Europe and accounted for heterogeneity in Alzheimer's disease presentation by including participants across the cognitive continuum (from subjective cognitive decline to mild dementia), with varied demographic, clinical, and biomarker profiles. Stratified analyses by cognitive stage and CSF biomarker status were conducted to explore variability across at‐risk populations.

## CONSENT

All participants (or their legally authorized representatives) provided written informed consent before participation.

## Supporting information




**Supporting Information**: alz71462‐sup‐0001‐ICMJE.pdf

**Supporting Information**: alz71462‐sup‐0002‐SuppMat.docx
