# Supplementary material for: Speech‐based digital cognitive assessment for clinical trials: Detecting cognitive impairment stages and AD biomarker relations across European cohorts
Source: Alzheimers Dement. 2026 May 18;22(5):e71462. doi: 10.1002/alz.71462 (PMC13183597; doi:10.1002/alz.71462)
Supplement: Supplementary file 2 — Supporting Information: alz71462‐sup‐0002‐SuppMat.docx [file ALZ-22-e71462-s001.docx]

**Supplementary material**

Table 1. Study design overview

| **Timepoint** | **EPAD Scotland** | **β-AARC** | **BioFINDER-Primary Care** | **DELCODE / DESCRIBE** |
| --- | --- | --- | --- | --- |
| **T0** | ✔ (phone) | ✔ (phone) | ✔ (in-clinic, via app) | ✔ (phone) |
| **T3** | ✔ (phone) | ✔ (phone) |  | ✔ (phone) |
| **T6** | ✔ (phone) | ✔ (phone) | ✔ (phone) | ✔ (phone) |
| **T9** |  | ✔ (phone) |  | ✔ (phone) |
| **T12** |  | ✔ (phone) | ✔ (phone) | ✔ (phone) |
| **T15** |  |  |  | ✔ (phone) |

Table 2. Diagnostic classification procedures across cohorts

| **Cohort** | **Diagnostic categories used** | **Primary cognitive instruments** | **Diagnostic approach** | **Biomarkers used in diagnosis** |
| --- | --- | --- | --- | --- |
| β-AARC | CU, SCD | Clinical interview, subjective complaint | Clinician-based classification | No |
| EPAD | CU, MCI | CERAD, MMSE, CDR | Algorithmic + clinician consensus | No |
| DELCODE | CU, SCD, MCI, dementia | CERAD battery | Clinician consensus | No |
| DESCRIBE | CU, MCI | RBANS | Neuropsychological cut-offs | No |
| BioFINDER-Primary Care | CU, MCI, dementia | MMSE, CDR | Clinician diagnosis | No (biomarkers analyzed separately) |

Table 3. MCC values for SB-C biomarker classification across cohorts

| Dataset | Screening case | MCC |
| --- | --- | --- |
| β-AARC | Abeta | 0.27 |
|  | pTau | 0.29 |
| EPAD Scotland | Abeta | 0.24 |
|  | pTau | 0.22 |
| BioFINDER- Primary Care | Abeta | 0.33 |
|  | pTau | 0.44 |

Figure 1 Calibration plots

BioFINDER-Primary Care Abeta Calibration plot

BioFINDER-Primary Care pTau Calibration plot
